# Quality Index Charts of Al-Si-Mg Semi Solid Alloys Subjected to Multiple Temperatures Aging Treatments and Different Quenching Media

**DOI:** 10.3390/ma12111834

**Published:** 2019-06-06

**Authors:** Khaled Ahmed Ragab, Mohamed Bouazara, X.-Grant Chen

**Affiliations:** 1 Applied Sciences, University of Quebec at Chicoutimi, Chicoutimi, QC G7H2B1, Canada; mbouazar@uqac.ca (M.B.); xgrant_chen@uqac.ca (X.-G.C.); 2 Department of Metallurgy, Faculty of Engineering, Cairo University, Giza 12613, Egypt

**Keywords:** quality index charts, aluminum semi solid alloys, multi-temperatures aging, statistical design of experiments, TEM

## Abstract

The use of quality index charts is considered as an effective mean for evaluating the mechanical performance of Aluminum alloys for industrial engineering applications. The current study was carried out to investigate the influences of multiple-interrupted temperatures aging and quenching media (water versus air) on the quality index performance and precipitations evolution of A357 Aluminum semi solid alloys. Regarding the lack of similar investigations applied on such alloys, the quality index charts were generated for Al-Si-Mg semi solid castings based on its tensile properties. These charts are used to determine the quality index, in MPa, as a simple mean for compromising the strength and ductility together in one value using the Drouzy model. The multiple temperatures aging cycles were applied to improve the quality index values of Al-Si-Mg semi solid alloys for enhancing its characteristic and performance to resist the mechanical failures relating to automotive dynamic parts. The evolution of Mg_2_Si hardening precipitates, formed for specific thermal aging cycles, was investigated using transmission electron microscopy (TEM). The results obtained in this work revealed that the optimum quality index values were obtained by the application of T6-thermal under-aging treatment cycles. The regression models, using a statistical design of experiments, indicated that the optimum strength and high-quality index values were obtained by the application of interrupted thermal aging cycles, mainly C_2,3_-T6/T4/T7 conditions.

## 1. Introduction

The excellent combination of the strength and ductility of A357 (Al-Si-Mg) semi solid alloys is considered to be a key factor in selecting such castings for automotive engineering applications, e.g., suspension control arms. One of the main focuses of recent investigations on aluminum alloys is to reach a suitable compromise between metallurgical parameters to achieve optimum quality and the cost effectiveness. The quality of aluminum alloys is affected by several metallurgical parameters. Recently, the semi solid casting using Swirled Equilibrium Enthalpy Device (SEED) technique and the unconventional thermal aging cycles were applied to enhance the quality of A357 alloys [1,2,3,4,5]. The SEED casting technique induces optimum microstructure with globular Si particles and fragmentation of brittle Fe-rich intermetallics, e.g., β-Fe and π-Fe iron phases. Such microstructural changes positively affect enhancing the ductility of aluminum alloys, which in turn improve the quality of such materials. On the other hand, the application of unconventional thermal aging treatment cycles, mainly multi temperatures aging cycles of T4/T6/T7, can further induce optimum compromise values of strength and ductility by inducing different sizes of Mg_2_Si hardening precipitates [6,7,8]. The precipitation of nano-sized β-Mg_2_Si particles is considered as the main strengthening mechanism of Al-Si-Mg alloys. The sizes, shapes, and distributions of such precipitates are significantly identified using transmission electron microscopy (TEM). It was reported by a previous recent study [9], that the maximum strength of Al-Si-Mg alloys might be achieved when the microstructure contains a combination of well-dispersed coherent β″ and semi-coherent β′ precipitates formed during multi temperatures aging treatments. Such unconventional aging treatment cycles applied at different quenching media may also have a significant effect on the quality of the A357 semi solid alloys through a number of microstructural changes. These changes take place as a function of the designated thermal treatment parameters, e.g., specific temperatures and times [10,11,12,13,14,15]. On the other side, the quality of Al-Si-Mg alloys can be evaluated by the generation of quality charts using specific quality index models. 

The quality index of A357 semi solid castings plays a vital role in determining the specific metallurgical parameters required to fulfil reliable mechanical parts used in automotive engineering applications, mainly the suspension control arm. In this study, there are several parameters affecting the quality index of such alloys investigated, mainly casting technique, heat treatment parameters, and quenching media. The quality of an alloy can be determined using specific mathematical equations, where both strength and elongation values can be combined using a single quality index value, Q [16,17,18]. Quality charts generated using these equations can be used for selecting the optimum heat treatment conditions to obtain the appropriate mechanical properties and quality indices of alloys investigated. It has been noted that there is a lack of studies focusing on the effect of multi-temperatures aging treatment on the quality index of semi solid aluminum alloys. On the other hand, the improvement in the performance of Al-Si-Mg semi solid alloys and an understanding of its relationship to heat treatment parameters may be accomplished by applying statistical analysis using the design of experiments approach. The statistical design of experiments is considered a powerful approach for understanding and selecting a set of process variables, which are most important to the thermal aging process. It can determine at what level these variables must be kept to optimize the quality characteristic of interest. The statistical design of experiments, using regression analysis, has the advantage of investigating the interactions between the various variables involved in this study, although it requires a large number of experimental trial runs as the number of variables increases. The reported developments were related to the designs and devoted to defining a mathematical model applicable to the problem under investigation [19,20].

In the present work, the influences of multiple temperatures aging cycles and quenching media (water versus air) on the quality of A357 semi solid alloys with the help of quality index charts were investigated. The statistical design of experiments was used to establish the interactions between the process variables and the factors varied of selected levels. The precipitation of nano-sized Mg_2_Si hardening precipitates during aging cycles was characterized using a transmission electron microscopy for establishing a microstructure–property relationship of produced materials. The aim to develop materials for automotive parts with optimum characteristic and quality performance led to a rising interest in the industry of aluminum semi solid castings heat-treated using multi-temperatures aging.

## 2. Materials and Methods

The SEED semi-solid casting technique was used to prepare the materials of A357 aluminum alloy (Al-7%Si-0.6%Mg-0.1%Fe). The SEED technique is based on achieving rapid thermal equilibrium between the metallic container and the bulk of the metal by proper process parameter selection such as pouring temperature, eccentric mechanical stirring, and drainage of a portion of eutectic liquid. The molten metal of the desired composition is prepared in a silicon carbide crucible of electric resistance furnace and poured into a metallic vessel whose thermal mass is sufficient to cool the melt. The vessel and its contents are swirled at 200 rpm where the heat is extracted to achieve the desired liquid-solid mixture, and then a small portion of eutectic liquid is drained to produce a self-supporting semisolid slug that is formed under pressure. The SEED process is coupled with a high-pressure die casting (HPDC) press to produce standard samples of A357 semi solid alloys used for materials characterization.

**Figure 1:**
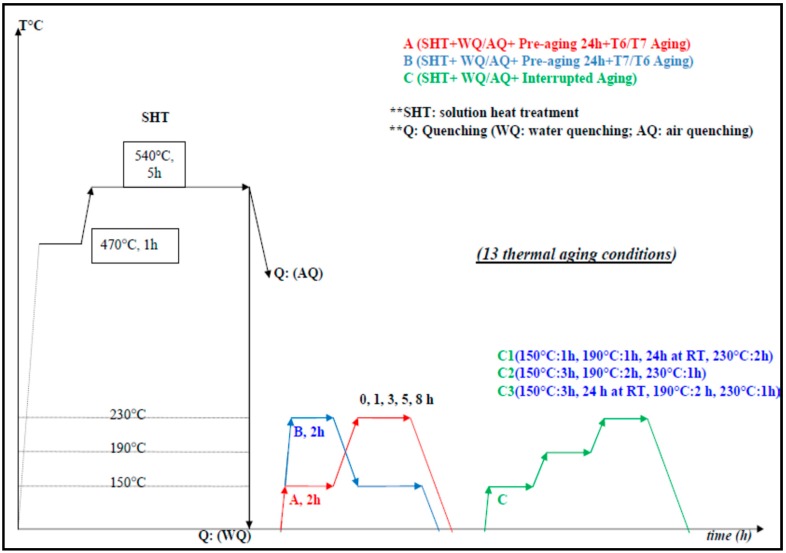
Multiple-Interrupted temperatures aging treatments employed for A357 semi solid alloys.

Various thermal cycles of different heat treatment conditions were applied using an electric resistance furnace of ±1 °C temperature deviation. As indicated by Figure 1, the samples were initially subjected to a two-step solution heat treatment process following by unconventional thermal cycles of multiple temperatures and interrupted aging treatment. The first step of the solution heat treatment, at 470 °C for 1 h, aims to dissolve the hardening Mg_2_Si phases into aluminum matrix and to avoid the incipient melting. The second step of the solution heat treatment is to fully dissolve primary Mg_2_Si and to fragment the Fe-rich intermetallic at 540 °C for 5 h. The samples were subsequently quenched in two different quenching media, mainly water medium at 60 °C and air quenching to the pre-aging ambient temperature. The thermal aging cycles are applied after having 24 h holding at ambient temperature, as indicated in Figure 1. In this work, three categories of unconventional thermal aging cycles were applied, mainly T6/T7 aging treatment (A cycles), T7/T6 aging treatment (B cycles), and T6/T4/T7 interrupted aging treatment (C cycles) with different aging times. These thermal treatment cycles were applied to have different levels of quality index values relating to the strength and ductility results obtained by standard tensile testing. After aging treatments, the tensile testing was carried out according to the standard dimensions of sub-sized specimens ASTM-E8M using a Servo hydraulic MTS Mechanical Testing machine (Instron, Norwood, MA, USA) [9]. The quality index charts were generated using the tensile properties by means of a mathematical model. The evolution of Mg_2_Si precipitates, during aging cycles, was investigated using a transmission electron microscopy (TEM, JEM-2100, JEOL, Tokyo, Japan) operated at 200 kV. The TEM samples, of 60 μm thickness and 3 mm diameter, were prepared by grinding and electro-polishing using a Twin-Jet device (EMS, 550D, Hatfield, PA, USA) in a nitric acid and methanol solution at −30 °C. The aim of TEM microstructure analysis is to indicate the relation between the quality index values and the precipitates characteristic of the Mg_2_Si strengthening phases concerning its size, density, and distribution for specific thermal aging conditions.

## 3. Results and Discussions

### 3.1. Quality Index Charts

The quality index of aluminum castings is considered as one useful tool in the selection of thermal aging parameters of the A357 semi solid alloys used for automotive dynamic parts as suspension control arm. The quality index of aluminum castings may be defined using numerical values correlated to the materials mechanical performance. These quality index values are presented by quality index charts that generate the relation between the strength and ductility values. In the present work, the quality index charts have been used concurrently with unconventional thermal aging cycles applied on aluminum semi solid alloys. These charts are generated to make it possible to determine the thermal aging parameters required for obtaining high-tensile properties and high-quality performance. The tensile results were evaluated using quality index charts derived from one model of quality index proposed by Drouzy et al. and cited by other studies [21,22,23]. The concept of quality index, Q, induced by Drouzy et al. [21] is to assess the quality of the alloy in terms of the tensile properties of Al-Si-Mg cast alloys, by generating iso-Q and iso-YS lines and subsequently constructing a quality index chart. The iso-Q lines and iso-YS lines were generated using the following equations:

Q = P_UTS_ + d log (S_f_), (1)

**Figure 2:**
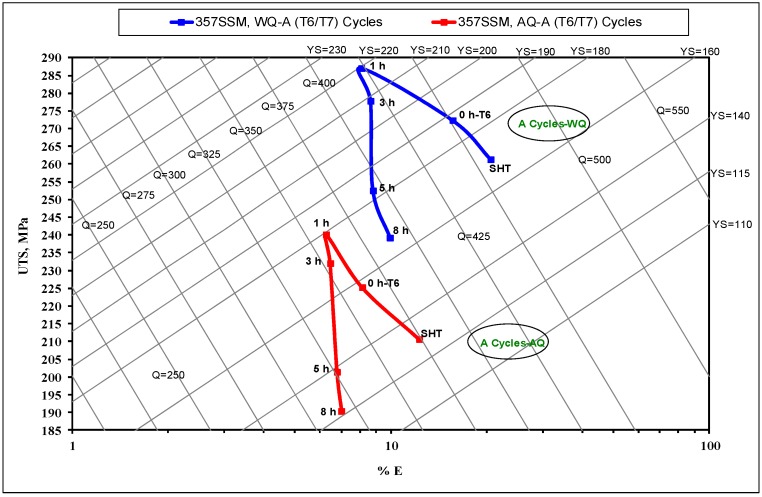
Quality index chart of A357-SSM heat-treated by multiple thermal aging treatments of T6/T7-A cycle with two quenching media.

**Figure 3:**
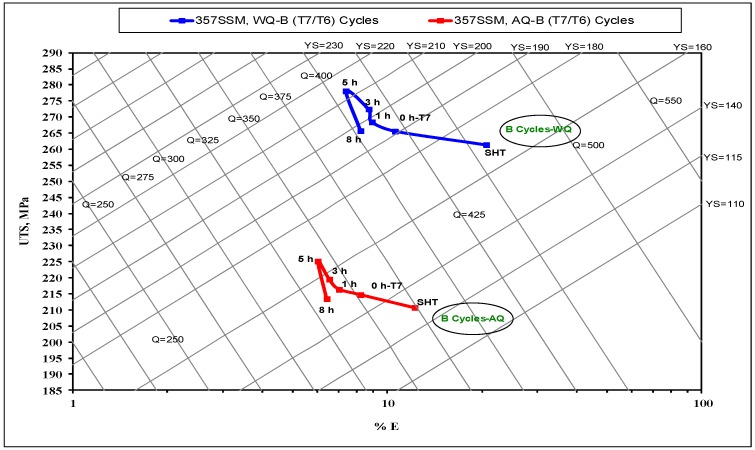
Quality index chart of A357-SSM heat-treated by multiple thermal aging treatments of T7/T6-B cycle with two quenching media.

**Figure 4:**
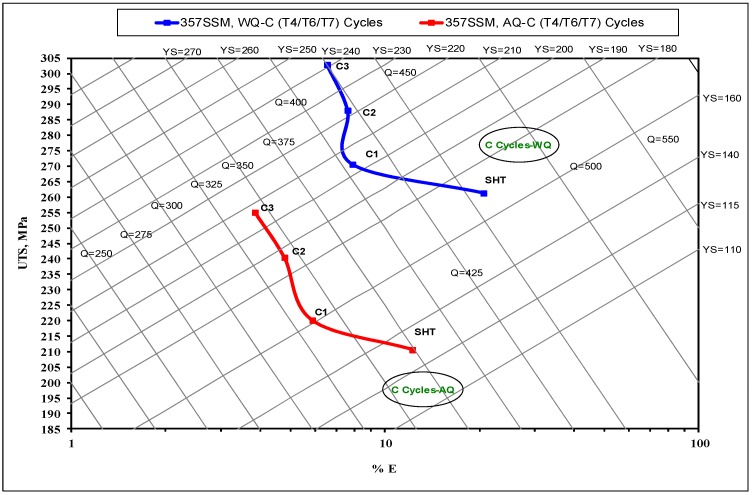
Quality index chart of A357-SSM heat-treated by interrupted aging treatments of T6/T4/T7-C cycle with two quenching media.

P_YS_ = a P_UTS_ − b log (S_f_) + C, (2)

where, Q is the quality index in MPa, P_UTS_ is the ultimate tensile strength in MPa, S_f_ is the elongation to fracture in pct., P_YS_ is the yield strength in MPa, and d is a material constant (d = 150). The parameters a, b, and c are material coefficients which are identified as 1, 60, and −13 respectively, for Al-Si-Mg alloys [21,22,23]. Figure 2, Figure 3 and Figure 4 show the influence of different aging cycles in two quenching media on the quality of A357 semi solid alloys. The applicable thermal aging cycles A, B, and C were investigated using quality index charts shown in Figure 2, Figure 3 and Figure 4, respectively. 

Regarding Figure 2, it was observed that the Q values were highly affected by the aging times of the second T7 aging step for the thermal treatment cycles of T6/T7-A conditions. The increase of aging times of T7 temper over 3 h leads to a significant decrease in Q values compared to those obtained after solution heat treatment (SHT), T6-A_0_ and 1 h of T6/T7-A_1_ conditions. This trend was observed for both quenching media of water and air. The high-quality value of 450 MPa, was observed for the under-aging T6-A_0_ condition. The increase of aging time results in a gradual decrease in Q values, from 425 MPa for 1 h to 387 MPa for 8 h. The decrease in quality values is related to the significant effect of T6/T7-A cycle on the strength and ductility values of A357 alloys. This effect is related to the increase in the strength up to the peak values after 1 h to 3 h of the second T7 aging step following by a significant decrease after 5 h to 8 h (over aging). On the other side, compared to the solution heat treatment (SHT) and T6-A_0_ conditions, the ductility decreases under the thermal aging conditions of A_1-8_, however, the ductility values slightly increased with increased aging times from 1 h to 8 h. Regarding Equation (1), it is noted that the quality index values (Q) are affected by both strength and ductility. Thus, the net effect of aging treatment cycles, A_1–8_, leads to a significant change in the Q values compared to SHT and A_0_ conditions. Concerning the quenching media effect, it was observed that the water quenching samples had higher Q values compared to those samples subjected to air quenching medium for all thermal aging conditions. In general, it should be noted that using water quenching results in a significant improvement in strength and Q values due to the fast cooling rate, as opposed to air quenching. However, the aged samples after air quenching ensure a reduction of residual stresses, as well as moderate ductility values at the expense of strength values [24,25]. This significant difference in quality index values is related to the higher strength values of water quenching samples. The application of the T7 aging step following to the T6 aging leads to a premature decrease in strength values after 1–3 h aging due to over aging. This over aging is attributed to the formation of coarse precipitates in the aluminum matrix at the high temperature of the T7 second aging step for T6/T7 A-cycle [24].

Figure 3 shows the response of quality index values to the application of T7/T6-B cycle thermal aging conditions. It is apparent that the quality index values are not significantly affected by the T7/T6-B cycle aging treatment compared to the influence of T6/T7-A cycle. Regarding the effect of the second T6 aging step for up to 8 h, the Q levels are quasi-parallel to the iso-*Q* lines, as is well-observed in the quality index chart. This trend illustrates that aging time up to the peak-strength does not affect the Q, which is related to the fact that the increase of the aging times up to 5 h results in a continuous increase in the strength at the expense of its ductility. This increase compensates for the reduction in ductility, in accordance with Equation (1). The significant point for such thermal aging cycle conditions is that the peak strength before over aging tends to be postponed until 5 h of the second T6 aging step. On the other hand, the solution heat-treated samples exhibited lower strength and higher ductility, resulting in a quality index value of 455 MPa, compared to those obtained by thermal aging conditions. The application of T6 temper following T7 aging leads to a significant improvement in the strength as well as moderated ductility and quality index values. The water quenching still shows a significant increase in both strength and quality index compared to air quenching. However, the ductility values are still not highly affected by the quenching media due to the reduction of residual stresses achieved by air quenching medium [25]. 

The influence of interrupted aging treatments (C cycle) on the quality index is displayed in Figure 4. The superior strength results are generally attained under such treatment conditions of interrupted aging. The high-strength value of 300 MPa and quality index of 425 MPa is achieved by the interrupted thermal aging condition, mainly the C_3_-cycle, which may be related to the formation of different sizes of Mg_2_Si hardening precipitates. The Q values show no significant difference between the aging conditions of C_2_ and C_3_ cycles. The application of such aging cycles leads to the formation of finer precipitates in the wide interspacing of coarse precipitates with high-density and good distribution in the matrix. These different sizes of nano-sized precipitates have a significant effect on the motion of dislocations resulting in a variation in strength and quality index values [26]. The quality index and strength results of water quenching are higher than those obtained by air quenching. It was reported [9] that the air quenching-aging revealed a negative effect on the evolution of Mg_2_Si precipitates. The microstructure analysis showed the formation of coarse Mg_2_Si phase out of the matrix, causing a decrease in the Mg and Si amounts required for the precipitation of nano-sized Mg_2_S particles during aging treatments [9]. 

Concerning the quality performance and economic factor, it was observed by the current study that the improvement in quality index and strength is achieved by the thermal aging conditions of A_0_-T6 aging and C_2,3_ interrupted aging cycles. Such thermal treatment conditions can be used as optimum aging parameters for particular engineering applications that require high values of quality index that compromise high strength and moderate ductility. The investigated A357 semi solid alloys are used in the fabrication of automotive dynamic parts, such as suspension control arms. The mechanical failure of such dynamic parts is mostly related to cracking issues that start from the aluminum matrix defects and propagate due to severe load conditions. The improvement of quality index values of such materials aims at enhancing the metal-matrix performance versus the crack propagation. The generation of such quality index charts facilitates the design and selection of metallurgical parameters appropriate for such applicable materials in the automotive industry.

### 3.2. Statistical Analysis 

The technique of design of experiments (DOE) for statistical analysis has been used to study the quality performance of A357 semi solid alloys subjected to various unconventional thermal aging cycles. In addition, it is used to develop regression equations for the response-dependant variables of quality index and tensile properties, which can be quantitatively analysed to better understand the effects of the different variables and their interactions. The strength (UTS) as well as Q values were analysed using Minitab software to obtain the regression models, the main effects plot, and the interaction plot, which indicate the relationship between the independent variables (thermal aging cycles) and the response (UTS and Q values) of the alloys investigated.

The regression Equations (3) and (4) were developed for Q values of A357 alloys, R^2^ (95.31% and 93.64% respectively), indicating the impact factor of tensile properties and thermal treatment parameters respectively, on the Q. The sign and magnitude of the coefficient values of the individual parameters significantly affect the response (Q). The positive coefficient denotes an increment in the Q owing to the associated increase in the individual parameters and their interactions. The magnitude value of the coefficient refers to the impact scope of these parameters and their interactions on the Q response.

Q = 78.4 + 1.05 UTS + 5.56%E,
(3)

Q = 276 + 71.3 Quenching Media + 0.0732 Aging Temperature (°C) − 4.89 Aging time (h),
(4)

Equation (3) shows the positive effect of ductility (E) on the quality index Q compared to strength (UTS), considering the applied thermal aging cycles. This may indicate the effect of thermal aging cycles on the strength and ductility regarding its impact on the quality index response. The mean Q values of A357 alloys are quite sensitive to the ductility variable factor. The regression Equation (4) indicates the significant effect of the quenching media variable on the Q compared to the thermal aging parameters applied. In addition, the negative effect of the thermal aging time of the second aging step is well-indicated on the Q values. Such regression equations show a satisfactory agreement with the quality index charts generated for A357 alloys subjected to various thermal aging cycles (Figure 2, Figure 3 and Figure 4).

**Figure 5:**
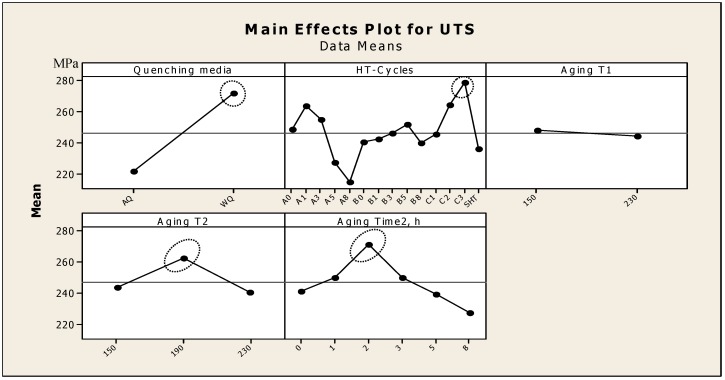
Main effects plot, using design of experiments (DOE), for the strength (UTS) (MPa) values of A357-SSM heat-treated using multiple thermal aging treatments.

Figure 5 indicates the main effects of the independent variables of the thermal aging parameters and quenching media, which affect the strength UTS values of A357 semi solid alloys. The plots of the main effects are characterized using average analysis of the raw tensile data to show the performance trend relative to the variations in the parameters studied. The values of each independent variable were compared within the variable range to observe its main impact on the UTS values. Whenever the line is horizontal, it signifies a slight impact of the independent variables on the UTS response. The optimal test parameters, with respect to these control variables, can be easily determined from the related plots. The water quenching medium shows higher strength values compared to the air quenching one. In addition, it indicated a significant change in the strength mean values with the various thermal aging cycles applied in this study. The significant peaks of strength were obtained by the application of the interrupted thermal aging conditions of C_2,3_ cycles. The increasing of the first aging step temperature (Aging T1), when the materials are aged to T7 at 230 °C, leads to a slight decrease in strength mean values. This decrease is normally related to the increase in the precipitates size formed at a higher aging temperature. On the other side, the application of second step aging (Aging T2) leads to a significant peak in the mean strength at an aging temperature of 190 °C for an aging time of 2 h. Such peaks of the strength may be related to the formation of smaller size precipitates, formed at 150 °C and 190 °C (B and C aging cycles), embedded into the inter-particle spacing of coarser Mg_2_Si, formed at 230 °C. 

**Figure 6:**
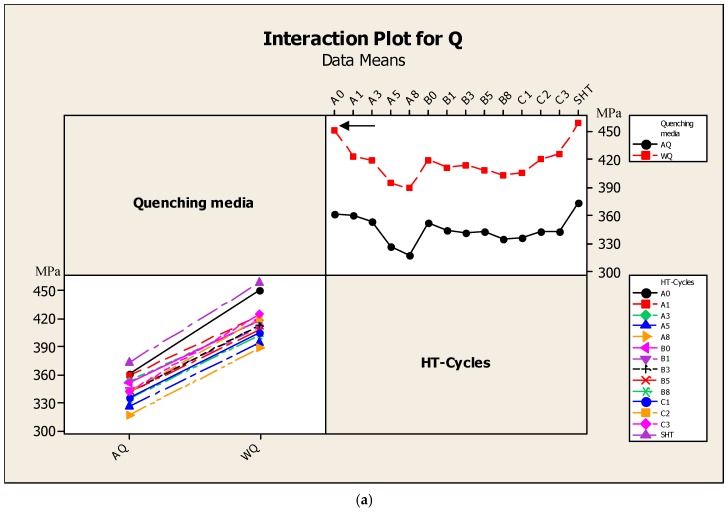

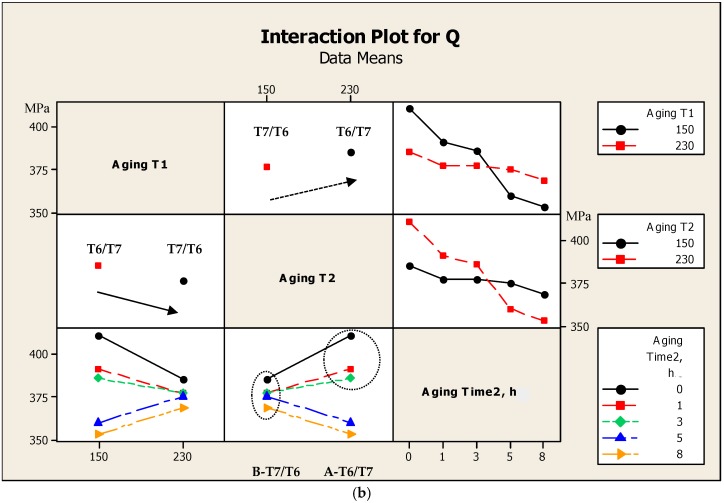
Interaction plot, using DOE, for the effect of (**a**) quenching media and heat treatment cycles and (**b**) aging parameters on the quality index, Q (MPa), values of A357-SSM heat-treated using multiple thermal aging treatments.

Figure 6 show the interaction plots of the independent variables applied in this study on the A357 semi solid alloys. Regarding the independent variables, Figure 6a indicates the dual effects of quenching media and heat treatment cycles (HT). While Figure 6b shows the dual effects of the thermal aging parameters, such as the first aging step temperature T_1_ (°C), second aging step temperature T_2_ (°C), and second aging step time, t_2_ (h). The interaction plots correlate the quality index Q with the parameters of multiple-interrupted temperature aging cycles and the quenching media. The water quenching (WQ) alloys show better quality values obtained for all of the independent variables compared to air quenching ones (AQ), as indicated in Figure 6a.

Regarding the various thermal treatment cycles, it has been recognized that the highest Q value was obtained after the application of the A_0_ aging cycle, 450 MPa, as indicated by the arrow in Figure 6a. Concerning the dual effects of the thermal aging parameters, the interaction plots in Figure 6b show that the application of dual aging temperatures T7/T6 resulted in a decrease in the mean Q values (indicated by the solid arrow in Figure 6b). On the other hand, the application of T6/T7 two aging-steps condition results in an increase in the Q values (indicated by the dotted arrow in Figure 6b). This explains the dual effects of aging temperatures, T6/T7-B cycles thermal aging condition, on the improvement of Q values. Regarding the dual effects of aging times and temperatures (t_2_ and T_2_), it is generally observed that the quality values decreased gradually with increasing aging times t_2_. However, the high-quality values were obtained after the application of T6/T7-A_0,1,3_ cycles for 0 h, 1 h. and 3 h of T7 aging step-times compared to the T7/T6-B_0,1,3_ cycles (indicated by the dotted circles in Figure 6b). This difference in quality index values of A357 alloys, accompanying the T6/T7-A_0,1,3_ cycles, is related to the coarsening of the precipitates at the expense of the small ones formed after T6 first stage [9]. Such a precipitates feature reduces the resistance to dislocation motion through the metal matrix and lead to increases in the ductility values, which have a significant impact in increasing the quality index values according to the regression Equation (3). Other studies [27,28] have investigated the effect of multi temperatures aging on the mechanical properties of aluminum alloys. It was revealed that the application of multi temperatures aging induced a slight positive effect on the strength values of aluminum alloys compared to the standard T6 aging treatment, mainly 354-type (Al-Si-Cu) and 6061 (Al-Mg-Si) of casting and wrought aluminum alloys, respectively. In this study, the strength values of A357 (Al-Si-Mg) semi solid castings were significantly enhanced by the application of multi temperatures aging treatments (280 MPa for C_3_ cycle) compared to the standard aging treatment (245 MPa for A_0_ cycle), as shown in Figure 5.

### 3.3. Precipitates Evolution

**Figure 7:**
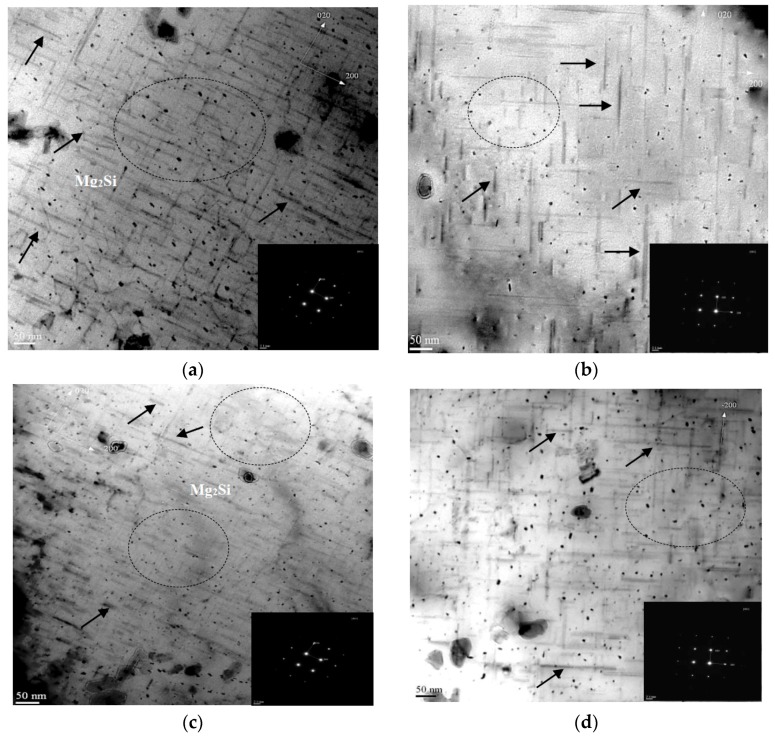
TEM images indicating the characteristics of Mg_2_Si precipitates of A357 semi solid alloys subjected to specific thermal aging conditions of A_3,_ A_5_, C_1_, and C_2_ Cycles. (**a**) Two-steps thermal aging cycle, A_3_; (**b**) Two-steps thermal aging cycle, A_5_; (**c**) Interrupted thermal aging cycle, C_2_; (**d**) Interrupted thermal aging cycle, C_1_.

The size and density of strengthening precipitates formed at specific thermal aging conditions of A_3,5_ and C_1,2_ cycles, for A357 semi solid alloys are shown in Figure 7. The fine precipitates of nano-sized Mg_2_Si are formed due to the application of thermal aging cycles. Such thermal aging conditions of A and C cycles lead to the formation of various sizes and density of well-distributed, strengthened Mg_2_Si phases relating to the specific applicable aging conditions.

The TEM microstructures indicate variations in the sizes of the formed nano-sized Mg_2_Si precipitates. A quantitative analysis using a software program connected to an optical microscopy was used to measure the size of such precipitates, indicated in the TEM micrographs. The sizes of precipitates range from 26.18 ± 1.5 nm to 240 ± 1.7 nm and from 15.75 ± 1.1 nm to 210 ± 1.6 nm for aging cycles C_1_ and C_2_, respectively. On the other side, the size of Mg_2_Si precipitates for aging cycles of A_3_ and A_5_ ranges from 20.34 ± 0.5 nm to 221 ± 1.2 nm and from 35.18 ± 0.8 nm to 250 ± 1.4, respectively. Such precipitates of various Mg_2_Si sizes may be related to the formation of finer and fine phases of β″ and β′, respectively. Such black strengthened particles are identified as Mg-Si-containing precipitates according to its shape, sizes, and electron diffraction patterns of [001] and [010] Miller indices directions. The diffraction electron patterns, shown for each TEM micrograph, were used to identify the Mg_2_Si precipitates according to its crystallographic orientations. The microstructures revealed the Mg_2_Si as spherically-shaped particles of vertical *z*-direction (indicated by dotted circles) and of rod/needle-shaped (indicated by dotted circles). Such spherical Mg_2_Si particles were also observed in other studies [9,29,30]. The two precipitates size types, that may be identified mainly as β″ and β′, are related to the application of multiple and interrupted thermal aging cycles, mainly A, C cycles. Regarding Figure 7a,b for thermal aging conditions of T6/T7-A_3,5_ cycles, it significantly indicated the formation of coarser and less-dense precipitates for A5 thermal aging cycles compared to such precipitates sizes and density obtained for A_3_ aging condition. This difference in the sizes and density of strengthen precipitates are related to increasing the aging time of T7 second aging stage from 3 h to 5 h for aging cycles A_3_ and A_5_, respectively. This may explain the trend drop in strength and quality index values as indicated in the quality index charts and statistical analysis applied for such specific thermal aging conditions of A_3,5_ cycles. On the other hand, Figure 7c,d indicate that the application of interrupted thermal aging conditions of C_2_ cycle leads to the formation of finer and denser Mg_2_Si precipitates compared to those formed by the application of A_3,5_ thermal aging cycles. This may also explain the highest strength and moderate quality index results for C_2_ thermal aging cycles compared to A_3,5_ cycles, as indicated in Figure 2, Figure 5 and Figure 6. Such finer and denser precipitates, formed by the C_2_ thermal aging cycle, impede the movement of dislocations through the matrix, enhancing the strength of the materials investigated. On the contrary, by the formation of coarse-widely spaced precipitates, the alloy loses its strength properties due to dislocation bowing (over looping) mechanisms leading to the earlier yielding of the alloy [30]. In a previous work [9], it was indicated that the C_3_ aging condition showed the highest density of well-distributed precipitates of Mg_2_Si compared to B_5,8_ aging cycles. In addition, the formation of various sizes of intermediate semi coherent phases may contribute to alloy hardening with moderate ductility values to have high-quality index results, as indicated by the application of interrupted thermal aging conditions of C_2,3_ cycles.

## 4. Conclusions

1. The water quenching alloys showed better quality index values obtained for all thermal aging cycles compared to air quenching ones.
2. The application of multiple thermal aging parameters of T6/T7-A_1,3,5,8_ cycle conditions indicates a significant negative effect on the UTS strength values (for T7 aging times of 1 h–8 h) compared to the aging conditions of B and C cycles.
3. The optimum quality index values obtained by this study were achieved by the application of the aging condition of A_0_-T6 cycle and C_2,3_-interrupted aging cycles. This may be related to the ductility enhancement of alloys investigated for such thermal treatment conditions.
4. With regard to regression models for quality results, the mean quality values of A357 alloys are significantly sensitive to quenching media compared to other independent variables of thermal aging parameters. In addition, the ductility variable had a significant positive impact on the quality index values compared to the strength factor. This may explain the highest quality values after thermal aging condition of T6-A_0_ cycle.
5. With respect to the statistical analysis using DOE for thermal aging parameters, the optimum mean quality index values were obtained by the application of the aging conditions of A_0_ and C_3_ cycles. The interrupted thermal aging cycles of C_2_ and C_3_ arrive at the optimum UTS strength (285–300 MPa) and high-quality index (425 MPa) compared to the other aging parameters applied in this study.
6. The application of interrupted aging conditions of the C_2_ cycle led to the formation of finer and denser precipitates of various sizes compared to the two-steps aging of A_3,5_ cycles. Such finer precipitates of various sizes induced a significant increase in the strength and quality index values for thermal aging cycles of C conditions.
